# Improving prehospital trauma management for skiers and snowboarders - need for on-slope triage?

**DOI:** 10.1186/1752-2897-5-5

**Published:** 2011-04-26

**Authors:** Rebecca M Hasler, Uli Schmucker, Dimitrios S Evangelopoulos, Ron E Hirschberg, Heinz Zimmermann, Aristomenis K Exadaktylos

**Affiliations:** 1 Dept. of Emergency Medicine, University of Bern, Inselspital, Bern, Switzerland; 2 Dept. of Trauma and Reconstructive Surgery, University of Greifswald, Greifswald, Germany; 3 Dept. of Orthopedic Surgery, University of Bern, Inselspital, Bern, Switzerland; 4 Dept. of Physical Medicine and Rehabilitation, Massachusetts General Hospital, Boston, USA

**Keywords:** skiing, snowboarding, triage, prehospital care, injury

## Abstract

**Background:**

Injuries from skiing and snowboarding became a major challenge for emergency care providers in Switzerland. In the alpine setting, early assessment of injury and health status is essential for the initiation of adequate means of care and transport. Nevertheless, validated standardized protocols for on-slope triage are missing. This article can assist in understanding the characteristics of injured winter sportsmen and exigencies for future on-slope triage protocols.

**Methods:**

Six-year review of trauma cases in a tertiary trauma centre. Consecutive inclusion of all injured skiers and snowboarders aged >15 (total sample) years with predefined, severe injury to the head, spine, chest, pelvis or abdomen (study sample) presenting at or being transferred to the study hospital. Descriptive analysis of age, gender and injury pattern.

**Results:**

Amongst 729 subjects (total sample) injured from skiing or snowboarding, 401 (55%, 54% of skiers and 58% of snowboarders) suffered from isolated limb injury. Amongst the remaining 328 subjects (study sample), the majority (78%) presented with monotrauma. In the study sample, injury to the head (52%) and spine (43%) was more frequent than injury to the chest (21%), pelvis (8%), and abdomen (5%). The three most frequent injury combinations were head/spine (10% of study sample), head/thorax (9%), and spine/thorax (6%). Fisher's exact test demonstrated an association for injury combinations of head/thorax (p < 0.001), head/abdomen (p = 0.019), and thorax/abdomen (p < 0.001).

**Conclusion:**

The data presented and the findings from previous investigations indicate the need for development of dedicated on-slope triage protocols. Future research must address the validity and practicality of diagnostic on-slope tests for rapid decision making by both professional and lay first responders. Thus, large-scale and detailed injury surveillance is the future research priority.

## Background

The popularity of alpine skiing and snowboarding has significantly increased over the last decades. Current sports technology allows for extreme racing manoeuvres at high-speed. Modern slope designs often demand substantial risk taking and advanced skills. Consequently, alpine sports range amongst the most injurious tourist activities in the Swiss Alps.

Previous studies reported typical injury patterns, the frequencies of specific injuries and their association with different slopes and terrains [1-12]. The hostile and outdoor alpine setting demands comprehensive resources in terms of skills, knowledge, technology and infrastructure. In general, standardized algorithms in prehospital and in-hospital care of the injured are discussed controversial [13-21]. Absence of dedicated standardized algorithms was previously associated with inadequate transfer patterns, increased morbidity, and transfer costs [19-21]. Nevertheless and to the best knowledge of the authors, dedicated on-slope triage protocols do not exist and evidence for the effectiveness and efficiency of comparable algorithms is missing.

The goal of this study was to understand the potential of a dedicated on-slope triage. A review of hospitalized skiing and snowboarding trauma cases was conducted to analyze characteristics of those in need for alpine emergency trauma care. These findings are discussed in the context of the current literature in order to identify future research priorities for the Bernese Oberland region.

## Methods

A six-year review of all consecutive serious injury cases of skiers and snowboarders documented in the hospital-based trauma data base (Qualicare Office, Medical Database Software, Qualicare AG, Bern, Switzerland) of a tertiary trauma centre in Switzerland (University Hospital Bern, Emergency Department) was performed. The inclusion criteria are depicted in table 1. Body regions and respective serious injuries according to ICD-10 classification are defined as follows: Head: skull fractures, brain injury (S02, S06). Spine: fractures, dislocations, spinal cord injury (S12-15, S22.0-1, S23.1, S24.0-2, S32.0-2, S33.1-2, S34.0-4). Thorax: rib and sternum fractures, haemo- and pneumothoraces, heart injuries, vascular injuries (S22.2-9, S27.0-S27.2, S26, S25). Pelvis: fractures, organ lesions, vascular injury (S32.2-5, S33.4, S35, S36, S37,). Abdomen: organ lesions, vascular injury (S35, S36, S37).

**Table 1:** Inclusion and exclusion criteria for study sample

Inclusion	Exclusion
Primary admission or transfer to study hospital	

Admission period July 1, 2000 - June 30, 2006	

Subjects aged ≥ 16 years	Subjects aged < 16 years

Major injury to one of the body regions: head, spine, chest, pelvis, abdomen	Isolated injury to the limbs

Injury due to skiing or snowboarding kinematics	Injury due to side activities (e.g. malfunctioning of lift, during break)

The main results reported are basic demographic variables (mean age, standard deviance, SD) as well as the frequency of specific serious injuries and injury patterns in multiple injury cases. Principal comparisons of injury combinations where made using the Fisher's exact test. Mean values were compared using the student-t-test. Statistical significance was set at p < 0.05.

## Results

The trauma data base identified 729 subjects with injuries from skiing or snowboarding, 576 (79%) of which were skiers and 153 (21%) snowboarders. A total of 401 (55%) suffered from isolated limb injury with snowboarders being more likely to suffer from isolated limb injury (n = 312, 54% versus n = 89, 58%), although not significant (p > 0.05). With respect to the purpose of the study, these were excluded from statistical analysis. The remaining 328 (45% of total sample) fulfilled the entry criteria (see table 1) and are henceforth referred to as "study sample". Women (66%) dominated over men (34%). Skiers were significantly older than snowboarders (mean age 40 years, SD ± 14.5 years, versus mean age 23 years, SD ± 7.5 years, p ≤ 0.01) Skiers and snowboarders did almost equally fulfil the entry criteria (45% versus 44%, p > 0.05).

The majority of subjects presented with monotrauma (n = 256, 78%) while the remaining n = 72 (22%) were diagnosed with multiple injuries (specific injuries at ≥ 2 body regions, see table 1). The mean Injury Severity Score (ISS) was documented as ISS 13 (SD ± 3.7, range 6 to 75). Two fatalities were reported (<1% of study sample). Injuries to the head and/or spine accounted for the largest proportion in the study sample (see table 2). In addition, table 2 presents the frequency of multiple injury by injury to a specific body region and the frequency of injury combinations. The latter showed associations for injury combinations to the head and thorax, head and abdomen, and thorax and abdomen. The most frequent injury combinations were head/spine (11% of study sample, 44% of multiple injured sample), head/thorax (9%, 43%), spine/thorax (6%, 26%), head/abdomen (3%, 13%), spine/abdomen (2%, 11%), chest/pelvis (2%, 11%).

**Table 2:** Frequency of injuries in the study sample and in the multiple injured subsample by injured body region, frequency of injury combinations by body regions

	Head	Spine	Thorax	Pelvis	Abdomen
**Study sample by injured body region: n (% of 328)**					

	171 (52)	142 (43)	68 (21)	25 (8)	18 (5)

**Multiple injured subsample: n (% of body region category)**					

	57 (33)	43 (30)	43 (63)	12 (48)	13 (72)

**Injury combinations: n of positive combination (% of study sample/% of multiple injured sample/% of those suffering from injury (in rows) amongst those suffering from injury (in column))**, **p-value of Fisher's exact test in total sample**					

***Head***	-	-	-	-	-

***Spine***	32 (10/44/19) 0.826	-	-	-	-

***Thorax***	31 (9/43/18) **<0.001**	19 (6/26/13) 0.076	-	-	-

***Pelvis***	6 (2/8/4) 0.633	8 (2/11/6) 0.122	5 (2/7/7) 0.074	-	-

***Abdomen***	9 (3/13/5) **0.019**	3 (1/4/2) 1.000	8 (2/11/12) **<0.001**	2 (1/3/8) 0.124	-

## Discussion

This article reviews demographic and injury data from a sample of skiers and snowboarders admitted to a tertiary trauma centre in Switzerland. In general, the documented distribution of injuries per body region is in accordance with previous reports [1,2,20]. The proportion of fatalities is comparably low, yet ranges amongst those published recently [3,20,21]. The age difference between skiers and snowboarders is well known and was found accordingly in the investigated study sample.

The strengths of this approach comprise the comparably large study sample and the standardized documentation in an established data base. However, the approach merits further discussion. The review is limited to the information provided by the data base. The sample itself is restricted to subjects suffering from injuries to the head and/or body stem which might comprise the external validity of the findings. Moreover, a selection bias is apparent due to the study hospital's prominent position as the leading referral centre within the Berne-Alps emergency care network. This mandated exclusion of subjects suffering from isolated limb injury; yet this group is transferred to the study hospital due to individual characteristics (i.e. severity) of their limb injury only. The authors, however, consider the approach presented as suitable for the purpose to understand basic characteristics of injured alpine sportsmen and to learn about the next steps on the way to an alpine triage protocol.

Globally, a broad variety of standardized trauma care algorithms is established, e.g. Advanced Trauma Life Support (ATLS) [17,18], Prehospital Trauma Life Support (PHTLS) [13,22], and International Trauma Life Support (ITLS) [15,23], all of which are integrated into regional air and ground emergency networks, logistic and technical providers, emergency call operators, hospitals, and other medical service providers. Most authors attribute improvement of trauma care performance or outcome indicators to continuous medical education [16], while trauma registries and other injury surveillance instruments are considered the backbone of quality management and scientific evaluation [24]. Nevertheless, and despite reported improvement of "soft" parameters (e.g. self-reported confidence, level of preparedness, knowledge) the scientific evidence for improved objective trauma outcomes through employment of such algorithms is still missing [13-15,17,18]. When the specific alpine setting is concerned, recent studies indicate a great disparity of medical training status amongst mountain rescuers [25]. Trauma care appears to be underrepresented in the surveyed training protocols [25], at least when compared to the proportion of trauma-calls among all mountain rescue calls that were documented elsewhere [21].

An Austrian study reports 12% unjustified helicopter transports and 10% unjustified ambulance transports in consequence of lacking field triage [20]. In this context, Küpper et al. emphasized the significance of on-scene suspicion of multiple injuries, which was the most frequent underdiagnosed injury category in a study sample considered representative for Swiss ski regions [19]. Almost all current prehospital and early in-hospital care algorithms contain triage-like primary assessments. Triage was initially developed for mass casualty incidents in military and civil wars. While triage-like procedures have already been employed in many mediaeval wars, the term triage refers to the "triage-transport-treatment-sequence" of the French Military Forces [26]. Current triage protocols are designed for mostly civil purposes and situations with an apparent mismatch of injured and resources to care for these injured. The Bern University Hospital's Emergency Department has fully adapted the ATLS^© ^protocol and all trauma patients are seen immediately by a certified ATLS^© ^provider (usually the consultant on call). After the primary survey patients are triaged towards resuscitation or department floor care. Emergency trauma care in an alpine setting, however, is not comparable to usual emergency medical services (EMS). Often, arrival on scene is prolonged e.g. due to delayed emergency calls and limited accessibility. The environment is hostile (e.g. low temperature, windstorm, snowfall) and the first responders usually have varying skills and experience (i.e. lay persons like the family, other skiers, ski patrollers). Usually the pist patrollers (e.g. local pist rescue services) are alerted and the patient transported to the nearest ski lift station by sledge. After arrival at the station, a rapid decision for either air transport or other modes (i.e. by ski lift or ski sledge transport to an ambulance car meeting point) is made.

Ideally, an on-slope triage algorithm manages to identify index injuries that trigger transport by adequate modes and to adequate target hospitals. Such procedures need to be simple, independent from advanced medical technology, and applicable by both EMS and non-EMS personnel under alpine outdoor conditions. Current knowledge on alpine emergency trauma care indicates three major challenges: 1. Hostile and random environment with limited options to follow established algorithms. 2. Extraordinary consumption of resources for a comparably small number of life threatening injuries. 3. Substantial impact of random presence of, and decision-making by, lay first responders or non-acadamic health care professionals on resource allocation. The authors strongly believe that a dedicated on-slope-triage algorithm can assist in improving quality of emergency care and in reducing costs of the services provided under these very specific conditions. This is said despite the ongoing controversy about the effectiveness, efficiency, and efficacy of trauma care algorithms, all of which are developed for and usually employed under vastly different conditions.

The authors suggest an on-slope scoring system that provides two scores. These trigger the subsequent decision-making on basically mode of transport and target hospital:

- (1) a status score that is weighted against a threshold score and releases an immediate cascade of action (level of urgency)

- (2) a prognostic score that indicates the probability of future escalation or de-escalation of treatment (level of preparedness)

The authors are convinced that this bimodal approach adequately respects the principles of modern civilian triage: allocation of priorities on all levels of care and throughout all management phases executed by means of thorough consideration of skills, knowledge, situation, resources, and foreseen consequences of any action including non-action.

In doing so, the state of consciousness is evidently a strong and easy to assess predictor for a broad range of severe traumatic and non-traumatic morbidities. As with other trauma scoring systems (e.g. Revised Trauma Score, RTS), the alpine scoring system must be heavily weighted towards the state of consciousness to compensate for major head injury, severe hypothermia, acute neurological and acute cardiovascular diseases in the absence of relevant multisystem morbidities. A comparable weighting is required for restricted airway and ventilation, peripheral and central neurological deficits. As far as unspecific co-morbidities and conditions are concerned, these must be integrated as a surrogate value, especially in case of borderline scores. Such conditions and morbidities comprise e.g. age, external bleeding, suspected or confirmed severe hypothermia. Nevertheless, further research is needed to further evaluate their respective predictive values. The same is true for the mechanisms of injury, i.e. fall, deep fall, crash to other person, crash to fixed object. Such criteria are used in those well established standardized algorithms mentioned above (i.e. ATLS^©^-criteria for emergency room treatment); however, these were not developed for the typical alpine setting. In fact, these criteria can assist in suspecting unstable pelvic injury, unstable spine injury, and multisystem injury [1,3,6,9,10,12,27] which have been shown to be frequently underdiagnosed and are, in general, challenging to determine prior to diagnostic imaging. In this context, further large-scale research that addresses the previously reported characteristics of skiers versus snowboarders and helmeted versus unprotected snows sportsmen is essential [1-3,5,6,8,10,12,27-29]. By all means, the goal of any on-slope triage algorithm is the targeted interpretation of physiologic, vital signs and respective predictors (i.e. demographic factors, injury mechanism) rather than to identify the underlying injury. In fact, the entire well established preclinical and early clinical emergency algorithms do strictly follow this philosophy.

Once a standardised on-slope triage algorithm exists, is has to be distributed in the skiing population, the health care professionals and those working in the skiing business. The authors suggest a distribution based on three columns: (1) provision of a mobile-phone-application (2) provision of a hotline-connection to a trained dispatcher (3) distribution of a paper-based version to all ski-pass holders (provision at point-of-sale, per download when booking tourist arrangements, etc.). If possible at the time of implementation, geotracking applications (i.e. GPS) can assist to locate the victim or the first responder respectively. In this context, mobile phone-based e-call-services are a promising development both in the automotive safety and the ambient assisted living sector. Their use has increasing constantly over the last years. The product can be easily modified for "push-the-red-button-in-case-of-emergency" use and is readily available at low costs. Such technology appears to be highly attractive for the young, educated, technophile snow sportsmen population. Irrespective of a subsequent triage algorithm, a regional e-call-system can significantly reduce the arrival time of the rescue team.

The Swiss Alps are especially attractive to skiers from abroad. Hence, the related cost compensation market for emergency care is highly fractioned, including off-pocket payment, private insurance, specific travel insurance, public national or European health insurance, and even social accident insurance or worker compensation fund insurance. This might limit the interest of individual Swiss insurance companies to invest in the development of care algorithms despite the potential of future savings. Hence, the authors strongly believe that alternative co-funding is required in the developmental stages. Since "safety" is a substantial add-on value to the virtual product called "Snow sports region Bernese Oberland", the existence of a comprehensive Alpine rescue network might be used for marketing purposes and consequently co-financed by regional development funds and regional businesses.

Although this paper focuses on the model of injured snow sportsmen, an interdisciplinary approach that covers the broad spectrum of emergencies as reported earlier [21], e.g. stroke, acute cardiovascular diseases, or acute hypoglycaemia, is required. Such will be subject to further investigations prior to piloting a triage algorithm for the Bernese Oberland Region.

## Conclusions

The data presented and those previously reported suggest an urgent need for further research in the field of prehospital alpine trauma and emergency management. Further investigations must focus on the specific resources required in alpine settings, outcome criteria such as emergency-call-to-needle-time, fatality, and morbidity, as well as cost effectiveness of any means applied. This requires large-scale pre-post-intervention-studies.

## Competing interests

The authors declare that they have no competing interests.

## Authors' contributions

Study concept and design: AE, HZ. Data collection: RH. Data analysis and interpretation: US, RH, RHi, DE, AE. Drafting of manuscript: RH, US. All authors reviewed the final draft of the manuscript.
